# Similar Viral and Immune Characteristics of Kaposi Sarcoma in ART-treated People Living With HIV and Older Patients With Classic Kaposi Sarcoma

**DOI:** 10.1093/ofid/ofae404

**Published:** 2024-07-12

**Authors:** Léna Royston, Aude Jary, Carolina A Berini, Tsoarello Mabanga, John Lin, Amélie Pagliuzza, Nicolas Chomont, Ivan V Litvinov, Alexandra Calmy, Valentin Leducq, Vincent Calvez, Anne-Geneviève Marcelin, Stéphane Isnard, Jean-Pierre Routy

**Affiliations:** Chronic Viral Illness Service, Department of Medicine, McGill University Health Centre, Montreal, QC, Canada; Research Institute of the McGill University Health Centre, Infectious Diseases and Immunity in Global Health Program, Montreal, QC, Canada; Division of Infectious Diseases, Geneva University Hospitals, Geneva, Switzerland; Sorbonne Université, INSERM, Institut Pierre Louis d'Épidémiologie et de Santé Publique, Assistance Publique—Hôpitaux de Paris (AP-HP), Hôpitaux Universitaires Pitié-Salpêtrière—Charles Foix, Laboratoire de Virologie, Paris, France; Chronic Viral Illness Service, Department of Medicine, McGill University Health Centre, Montreal, QC, Canada; Research Institute of the McGill University Health Centre, Infectious Diseases and Immunity in Global Health Program, Montreal, QC, Canada; Chronic Viral Illness Service, Department of Medicine, McGill University Health Centre, Montreal, QC, Canada; Research Institute of the McGill University Health Centre, Infectious Diseases and Immunity in Global Health Program, Montreal, QC, Canada; Chronic Viral Illness Service, Department of Medicine, McGill University Health Centre, Montreal, QC, Canada; Research Institute of the McGill University Health Centre, Infectious Diseases and Immunity in Global Health Program, Montreal, QC, Canada; Centre de Recherche du CHUM, Department of Microbiology, Infectiology and Immunology, Université de Montréal, Montreal, QC, Canada; Centre de Recherche du CHUM, Department of Microbiology, Infectiology and Immunology, Université de Montréal, Montreal, QC, Canada; Research Institute of the McGill University Health Centre, Infectious Diseases and Immunity in Global Health Program, Montreal, QC, Canada; HIV Unit, Division of Infectious Diseases, Geneva University Hospitals, Geneva, Switzerland; Sorbonne Université, INSERM, Institut Pierre Louis d'Épidémiologie et de Santé Publique, Assistance Publique—Hôpitaux de Paris (AP-HP), Hôpitaux Universitaires Pitié-Salpêtrière—Charles Foix, Laboratoire de Virologie, Paris, France; Sorbonne Université, INSERM, Institut Pierre Louis d'Épidémiologie et de Santé Publique, Assistance Publique—Hôpitaux de Paris (AP-HP), Hôpitaux Universitaires Pitié-Salpêtrière—Charles Foix, Laboratoire de Virologie, Paris, France; Sorbonne Université, INSERM, Institut Pierre Louis d'Épidémiologie et de Santé Publique, Assistance Publique—Hôpitaux de Paris (AP-HP), Hôpitaux Universitaires Pitié-Salpêtrière—Charles Foix, Laboratoire de Virologie, Paris, France; Chronic Viral Illness Service, Department of Medicine, McGill University Health Centre, Montreal, QC, Canada; Research Institute of the McGill University Health Centre, Infectious Diseases and Immunity in Global Health Program, Montreal, QC, Canada; CIHR Canadian HIV Trials Network, Vancouver, BC, Canada; Chronic Viral Illness Service, Department of Medicine, McGill University Health Centre, Montreal, QC, Canada; Research Institute of the McGill University Health Centre, Infectious Diseases and Immunity in Global Health Program, Montreal, QC, Canada; Division of Hematology, Department of Medicine, McGill University Health Centre, Montreal, QC, Canada

**Keywords:** cellular immunity, human herpesvirus 8, human immunodeficiency virus, humoral immunity, Kaposi sarcoma

## Abstract

**Background:**

Reemergence of human herpesvirus 8 (HHV-8)–induced Kaposi sarcoma (KS) in people living with HIV (PLWH) despite antiretroviral therapy (ART) poses a clinical challenge because they already have favorable CD4 T-cell numbers and undetectable viral loads. We observed that clinical presentation in PLWH on ART resembled classic KS found in older HIV-uninfected patients and hypothesized that immunosenescence may thus play a role in occurrence of KS on ART. We compared viral and immune factors implicated in the development of KS in ART-treated PLWH (HIV KS) and HIV-uninfected classic KS patients (cKS), compared to controls without KS (HIV Control, cControls respectively).

**Methods:**

Plasma, peripheral blood mononuclear cell, and skin tissues were obtained from 11 HIV KS and 11 cKS patients and 2 groups of age-matched controls.

**Results:**

HIV KS participants were younger than cKS (aged 53 vs 75 years). HHV-8 genotypes did not differ between groups. Despite the younger age and a lower CD4/CD8 ratio, activated, exhausted, and senescent T-cell frequencies were similar between HIV KS and cKS. Anti–HHV-8 immunoglobulin G levels were higher and circulating HHV-8 DNA lower in HIV KS compared with cKS. Circulating platelet-derived growth factors AA-BB and granulocyte colony-stimulating factors were higher in HIV KS We observed similar levels of HHV-8 DNA and PD-1 expression in skin lesions from HIV KS and cKS patients.

**Conclusions:**

Altogether, early immune senescence could be involved in the development of KS in ART-treated PLWH. Higher anti–HHV-8 immunoglobulin G levels could be linked with lower circulating viral load. Such insights should help developing therapeutical strategies to prevent development and treat KS in PLWH on ART.

Incidence of Kaposi sarcoma (KS) in people living with HIV (PLWH) dramatically decreased since the era of antiretroviral therapy (ART). However, the pathognomonic skin lesions of KS are reminiscent of 1 of the strongest social stigmas of the AIDS pandemic. In the past 2 decades, a growing number of reports of a new form of KS affecting ART-treated PLWH despite HIV viral control and immune reconstitution has arisen [1,2]. Although seldomly observed, emergence or reemergence of KS in well-controlled PLWH yields emotional discomfort for patients and remains a clinical management challenge with incomplete responses to local or systemic treatments [3]. Although ART-treated KS presents with a mild form of the disease, mechanisms associated with the development of this type of KS are puzzling because those PLWH have a restored CD4 T-cell count and do not present with other comorbidities while on long-term ART [1].

Kaposi sarcoma is also observed in individuals without HIV infection, in sub-Saharan Africa (endemic KS), in transplant recipients (iatrogenic KS), or in older men with Mediterranean or Jewish ancestry (classic KS [cKS]). KS in ART-treated PLWH and classic KS occur mostly in men and have a similar cutaneous indolent disease presentation with limited skin lesions and rare visceral involvement [4].

The causative agent of KS is the human herpesvirus-8 (HHV-8) also known as Kaposi sarcoma–associated herpesvirus, a member of the herpesvirus family. This virus establishes a chronic infection with a latent and usually asymptomatic stage, associated with transient lytic reactivations leading to infectious viral particles production. Seven subtypes of HHV-8 have been reported (A, B, C, D, E, F, and Z) based on sequence homology of the Open Reading Frame (ORF) K1, and are differentially distributed worldwide, with debated differences in pathogenicity [5].

Factors associated with reactivation and KS development are associated with age-related immune suppression, AIDS, or immunosuppressive drugs used for organ transplantations [6]. A low CD4/CD8 ratio, as a marker of immune activation, has been associated with KS in ART-treated PLWH [4, 7]. Hence, this form of KS may be linked to immune deficiency, although not related to low CD4 T-cell count. We hypothesize that HIV-induced immune senescence may lead to progressive reduction of HHV-8 control, precipitating development of KS at an earlier age compared to older men with cKS. In-depth comparisons of HHV-8–specific immune responses in PLWH and old patients with KS has not been carried out yet.

We aimed to explore the viral and immune characteristics in blood and tumor involved in KS development in ART-treated PLWH compared to HIV-uninfected people with cKS in a prospective cohort study.

## METHODS

### Inclusion Criteria and Enrollment

A total of 44 participants were included in this study: 22 ART-treated PLWH, of whom 11 had KS (HIV KS) and 11 without KS (HIV controls) matched in age and sex, and 22 people without HIV (PwoH) including 11 with cKS and 11 without KS (cControls) matched in age and sex. Patients with KS resulting from immune reconstitution inflammatory syndrome after ART initiation were excluded [8]. KS diagnosis was confirmed by pathologists from the McGill University Health Centre (MUHC) based on pathological findings with detection of HHV-8 by immunochemistry in skin/lesion samples. None had received previous treatment for KS in the past 12 months. PLWH were recruited from the Chronic Viral Illness Service of the MUHC in Montréal, Quebec, Canada. PwoH were recruited at the Chronic Viral Illness Service and at the benign hematology clinic of the MUHC.

### Cell Isolation

From blood samples, plasma and serum were isolated by centrifugation and kept at −80°C until used. Peripheral blood mononuclear cells (PBMCs) were isolated using Ficoll-density gradient (Stemcell Technologies, Vancouver, BC, Canada) and kept in liquid nitrogen until used.

Skin/tumor punch were obtained from 7 HIV KS, 3 patients with cKS, and 2 HIV controls without KS. Biopsies were cut into even pieces; some were flash frozen in liquid nitrogen and kept at −80°C until used. Some pieces were fixed in formalin (ThermoFisher, Waltham, MS, USA) for 24 hours, transferred in 70% ethanol, and included in paraffin. Pieces were cut in smaller portions and subjected to 3 rounds of Liberase (Roche, Laval, QC, Canada). Mechanical lysis was performed between rounds by aspiration with needle and syringes. Cells were washed in RPMI containing fetal bovine serum (FBS) and antibiotics and kept cold until used.

### Quantitative Serology Assay

We have adapted a previously described HHV-8 serology assay [9]. Briefly: BCBL-1 cells (ATCC #ARP-3233), a B-cell line infected by HHV-8 but not Epstein-Barr virus (EBV), were stimulated with phorbol myristate acetate and incubated with plasma, then anti-human IgG fluorescent antibodies. Flow cytometry detected the frequency of cells positive for human IgG (detailed protocol in Supplementary materials).

### Detection of HIV and HHV-8 in Plasma, Peripheral Blood Mononuclear Cells and Skin Tissues

HIV DNA (total and integrated) and cell-associated RNA (LTR-gag) were quantified as previously shown in CD4 isolated from PBMCs (Stemcell Technologies) [10].

HHV-8 viral load was quantified by digital-droplet polymerase chain reaction (ddPCR) using an in-house protocol. Plasma viral RNA/DNA or DNA from PBMC or skin tissues were extracted using commercial kits (Qiagen, Toronto, ON, Canada) and kept at −80°C until used. Plasma RNA/DNA was used directly. Before ddPCR, genomic DNA from PBMCs or skin tissues were subjected to DNA digestion to increase droplet formation: 1 µg of DNA was incubated with 800 U/mL of HindIII enzyme (New England BioLabs Inc., Whitby, ON, Canada) in the supplied buffer at 37°C for 1 hour, followed by heat inactivation of the enzyme at 80°C for 20 minutes according to supplier's instructions.

The ddPCR was performed according to supplier's instructions (BioRad Laboratories, Montreal, QC, Canada). A set of primers and probe targeting HHV-8 ORF9 previously published and validated has been used (Supplementary Table 1) [11]. Copies of HHV-8 were normalized using copies of RRP30 divided by 2 (because 2 copies of the gene are present per cell). Viral loads obtained with our ddPCR assay from PBMCs and skin tissues were compared to viral loads quantified with a published and validated real-time quantitative PCR assay [12].

### Cell Phenotyping

Frozen PBMCs were thawed in RPMI containing 10% FBS, 100 U/mL of penicillin, and 100 µg/mL of streptomycin (thereafter referred as complete medium), washed with cold phosphate-buffered saline (PBS) and kept at 4°C or on ice until the end of the experiment. Cells were washed with PBS containing 2% FBS 1 mM EDTA (hereafter referred to as cytometry buffer). Skin cells were kept cold in cytometry buffer. PBMCs or skin cells were split at 10^6^ per condition and 5.10^5^ cells for controls and incubated with antibody cocktails for 20 minutes in the dark at 4°C. Panels were designed to assess T-cell and myeloid cells/dendritic cell function (list of antibodies provided in Supplementary Table 2). Cells were then washed in cytometry buffer and resuspended in PBS containing 2% paraformaldehyde until analysis. Cytometry acquisition was performed on a BD-Fortessa X20, analysis was performed using FlowJo version 10.9 (gating strategies described in Supplementary Figures 1, 2, 3).

### Genotyping of HHV-8

Twenty-nine extracted DNA were subjected to whole-genome sequencing to determine the HHV-8 genotype, as previously reported [13] (detailed methods in Supplementary material). All sequences were added to GenBank database (NIH, Bethesda, MD, USA; accession numbers: Supplementary Table 3).

### T-cell Function

T-cell response to HHV-8 was assessed by ELISPOT. PBMCs were thawed and rested overnight in complete medium. A total of 250 × 10^3^ PBMCs per well were left unstimulated (in complete medium) or incubated with selected peptides pools from LANA-1 protein or peptides covering the entire K12 protein at 5 µg/mL/peptide (JPT, Berlin, Germany; Supplementary Table 4) for 24 hours at 37°C. IFN-γ ELISPOTs (Mabtech, Nacka Streng, Sweden) were performed following supplier recommendations. Spot counting was performed on a CTL Immunospot reader. Results are presented as number of spots per condition minus spots in the unstimulated condition. Anti-CD3 antibody stimulation was used as positive controls and yield to a saturated signal for all participants.

### Cytokines and Biomarkers Plasma Levels

A 41-plex multiplex from Millipore (Burlington, MA, USA) was used to quantify cytokines, chemokines, and growth factors plasma levels. Plasma levels of soluble urokinase plasminogen activator receptor, growth differentiation factor 15, fibroblast growth factor 21, and intestinal fatty acid binding protein were quantified using enzyme-linked immunosorbent assay (ELISA; R&D Systems, Minneapolis MN, USA). Lipopolysaccharide levels were quantified by ELISA (Cusabio, Wuhan, China). Suppliers’ recommendations were followed for ELISA and multiplexes.

### Statistics

GraphPad Prism 9.3.0 (GraphPad, La Jolla, CA, USA) and SPSS 24.0 (IBM SPSS, Chicago, IL, USA) were used for statistical analyses. The statistical significance of differences between groups was assessed using nonparametric Kruskal–Wallis tests with Dunn's posttests or parametric *t*-tests, when appropriate. An α level of 5% was considered statistically significant (*P* value).

## RESULTS

### PLWH With KS Were Younger and had Lower CD4/CD8 Ratio Than PWoH With KS

HIV KS were younger than cKS (average, 53 vs 75 years of age, *P* < .001, Table 1). Because participants without KS were matched for age and sex, a similar difference was observed in the 2 control groups without KS. Clinical presentation of the disease was similar between the 2 groups of KS patients, sharing limited KS skin lesions, predominantly on the lower limbs (Supplementary Figure 4).

**Table 1.** Clinical Characteristics of Study Participants (n = 44)

Characteristics	HIV KS (n = 11)	HIV Controls (n = 11)	*P* Value HIV+	cKS− (n = 11)	CControls (n = 11)	*P* Value HIV–	*P* Value HIV KS vs cKS
Age, y	…	…		…	…		
Mean (SD)	53 (8.4)	53 (8.2)	1.00	75 (8.5)	75 (6.4)	1.00	<.001
Range	38–65	35–60		59–89	69–89		
Sex, no. (%)	…	…		…	…	…	.15
Male	11 (100%)	11 (100%)	1.00	9 (82%)	9 (82%)	1.00	
Female	0	0	1.00	2 (18%)	2 (18%)	1.00	
MSM, no. (%)	11 (100%)	11 (100%)	1.00	3 (27%)	3 (27%)	1.00	<.001
Diabetes, no. (%)	1 (9%)	3 (27%)	.29	1 (9%)	1 (9%)	1.00	1.00
Hypertension, no. (%)	1 (9%)	2 (18%)	.56	2 (18%)	3 (27%)	.63	.56
Statin use, no. (%)	1 (9%)	2 (18%)	.56	3 (27%)	2 (18%)	.63	.29
Current/former smoker, no. (%)	4 (36%)	6 (55%)	.42	3 (27%)	3 (9%)	1.00	.67
CMV seropositivity, no. (%)	11 (100%)	11 (100%)	1.00	9 (82%)	9 (82%)	1.00	.15
CD4 T cells/µL median (range)	621 (114–1099)	585 (398–967)	.79	416 (46–730)	897 (518–1271)	<.001	.11
CD8 T cells/µL median (range)	507 (379–1919)	473 (224–1116)	.17	339 (200–582)	415 (186–948)	.22	.04
CD4:CD8 ratio median (range)	0.84 (0.34–1.25)	1.16 (0.48–2.30)	.05	1.72 (0.75–1.90)	1.9 (1.06–6.08)	.09	.008
HIV viral load, log10 copies/mL	<1.7	<1.7	1.00	N/A	N/A	N/A	N/A
HIV duration (y) Median (range)	12.9 (2.8–28.7)	16.7 (2.2–34.0)	.27	N/A	N/A	N/A	N/A
ART use, no. (%)	11 (100%)	11 (100%)	1.00	N/A	N/A	N/A	N/A
PI use, no. (%)	0	0	1.00	N/A	N/A	N/A	N/A
ART duration, y	12^a^	16	.26	N/A	N/A	N/A	N/A
Anti-HHV8 IgG positive, no. (%)	11 (100)	10 (91)	.31	11 (100)	1 (9)	<.01	1.0

Abbreviations: ART, antiretroviral therapy; CMV, cytomegalovirus; KS, Kaposi sarcoma; MSM, men who have sex with men; N/A, not applicable or not available; PI, protease inhibitor.

^a^Data not available for 1 participant.

All HIV KS and HIV controls participants were men who have sex with men (MSM), whereas 9/11 (82%) were men including 3/11 (27%) MSM in the cKS and cControls groups (Table 1). No difference among comorbidities was found among groups.

Circulating CD4 T-cell counts at KS diagnosis were similar between PLWH with and without KS, conversely cKS had significantly lower CD4 T-cell counts than their cControls (*P* < .001, Table 1). CD4 T-cell counts were not significantly different among the 2 KS groups. As expected, CD8 T-cell counts were higher in HIV KS compared to cKS (*P* = .04). The CD4/CD8 ratio was then lower in HIV KS compared to cKS (*P* < .001) and was also lower in HIV KS compared to their HIV control group (*P* = .05). All PLWH had undetectable plasma HIV viral loads and none of them had been using protease inhibitors at the time of sampling.

### PLWH With KS had Lower Circulating HHV-8 DNA Than PWoH With KS Measured by ddPCR

**Figure 1.**
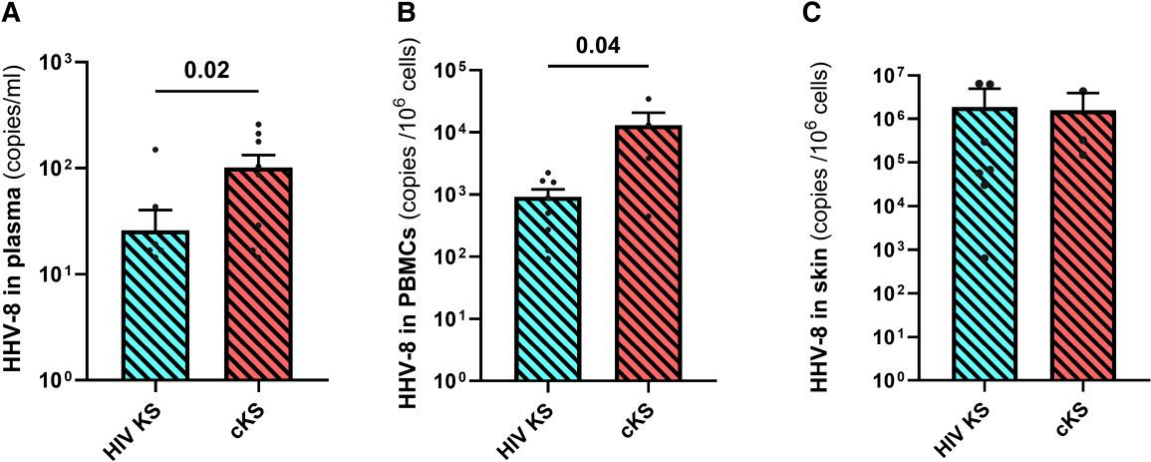
HHV-8 DNA quantification in blood and skin tissues. HHV-8 DNA was quantified in plasma (*A*), PBMCs (*B*), and skin tissues (*C*) samples with our in-house ddPCR assay in participants with KS. Only significant (<.05) *P* values are indicated. cKS, classic KS; KS, Kaposi sarcoma.

Using our in-house ddPCR assay (compared with published protocol in Supplementary Figure 5), HHV-8 DNA was detected in 19/22 patients with KS, either in plasma, PBMCs, or skin tissues. HHV-8 DNA levels in plasma and PBMCs were lower in HIV KS compared to cKS (*P* = .02, Figure 1*A*, and *P* = .04, Figure 1*B*, respectively). However, HHV-8 DNA levels were similar in skin biopsies between the 2 groups (Figure 1*C*). Higher HHV-8 levels were found in skin tissues compared to PBMCs or plasma.

HIV KS tended to have a higher number and frequency of HIV viral load blips and HIV RNA levels in CD4 T cells compared to HIV controls (Supplementary Figure 6).

### Phylogenetic Analysis Reveals Similar HHV-8 Subtypes in Both KS Groups

Sequencing and typing were effective for 15/19 (79%) of the HHV-8 PCR-positive KS patients, either by NGS or K1 sequencing by Sanger; we obtained 8 HHV-8 whole genomes, 2 HHV-8 ORF-K1m and 3 HHV-8 (ORF-K1) VR1. Based on the K1 and VR1-region phylogenetic analysis, 5 participants had an HHV-8 strain identified as subtype A (including 3 A5), 1 as subtype B, 5 as subtype C (including 3 C3), and 2 as a potential new variant of subtype D (Supplementary Table 3).

HHV-8 whole genome showed a majority of subtypes A/C (6/8), 1 sequence with subtypes B (identified as A5 with K1 analysis) and 1 with variant F1 (identified as B with K1 analysis). The potential new D variant clustered alone, no whole-genome sequences of E and D subtypes being available in GenBank database.

In total, distribution of HHV-8 types was mainly associated with patients’ origin rather than to clinical presentation or other epidemiological factors.

### In PLWH With KS Compared to PWoH With KS, Anti-HHV-8 IgG Levels Were Higher While Sharing Similar HHV-8-specific T-cells Frequency

**Figure 2.**
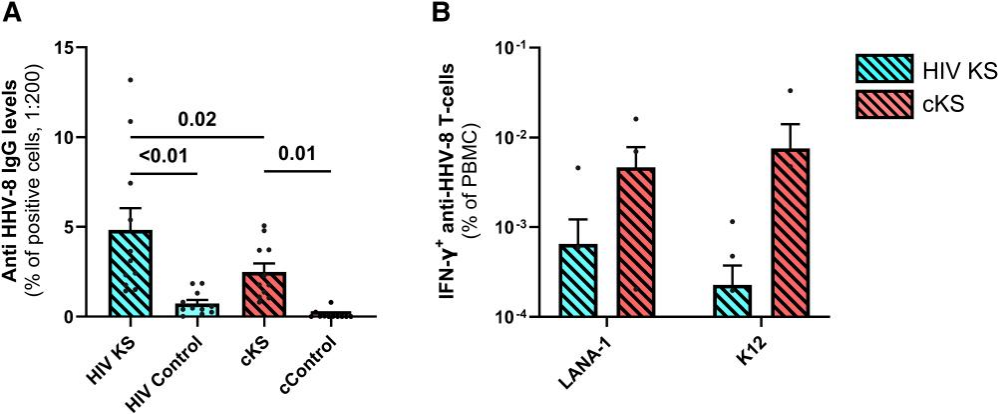
Specific anti-HHV8-8 immune responses in blood. Anti–HHV-8 IgG levels were compared between groups (*A*). Briefly, an in-house assay was designed using stimulated BCBL-1 HHV-8–infected cells incubated with participants’ plasma samples, and quantification of anti-human IgG-bound cells by cytometry. T-cell response to HHV-8 (*B*) was assessed by IFN-γ ELISPOTS after incubation with peptide pools for LANA-1 or K12 proteins and compared among groups. Only significant (<.05) *P* values are indicated. cKS, classic KS; KS, Kaposi sarcoma.

Anti–HHV-8 IgG levels were not detected in most cControls (10/11). All PLWH participants except 1 had detectable levels of anti–HHV-8 IgG with HIV KS having higher levels than HIV controls (*P* < .01). As expected, all cKS patients had detectable levels of anti–HHV-8 IgG compared to cControls (*P* < .01, Figure 2*A*). Interestingly, HIV KS had higher levels of anti–HHV-8 IgG than cKS (*P* = .02).

Anti-EBV IgG levels were higher in HIV KS compared to HIV controls. A similar tendency was observed between cKS and cControls. However, levels of IgG specific for another herpes virus, cytomegalovirus (CMV), were similar between KS groups (Supplementary Figure 7).

HIV KS and cKS had a very low and similar anti–HHV-8 T cells frequency in blood, both against LANA-1 (*P* = .32, Figure 2*B*) and K12 (*P* = .08, Figure 2*B*). Of note, K8.1 (3 peptides) and gpB (2 peptides) were also tested but did not induce detectable T-cell response.

### PLWH and PWoH With KS had Similar Levels of T-cell Activation, Senescence and Exhaustion Markers in Blood and Skin

**Figure 3.**
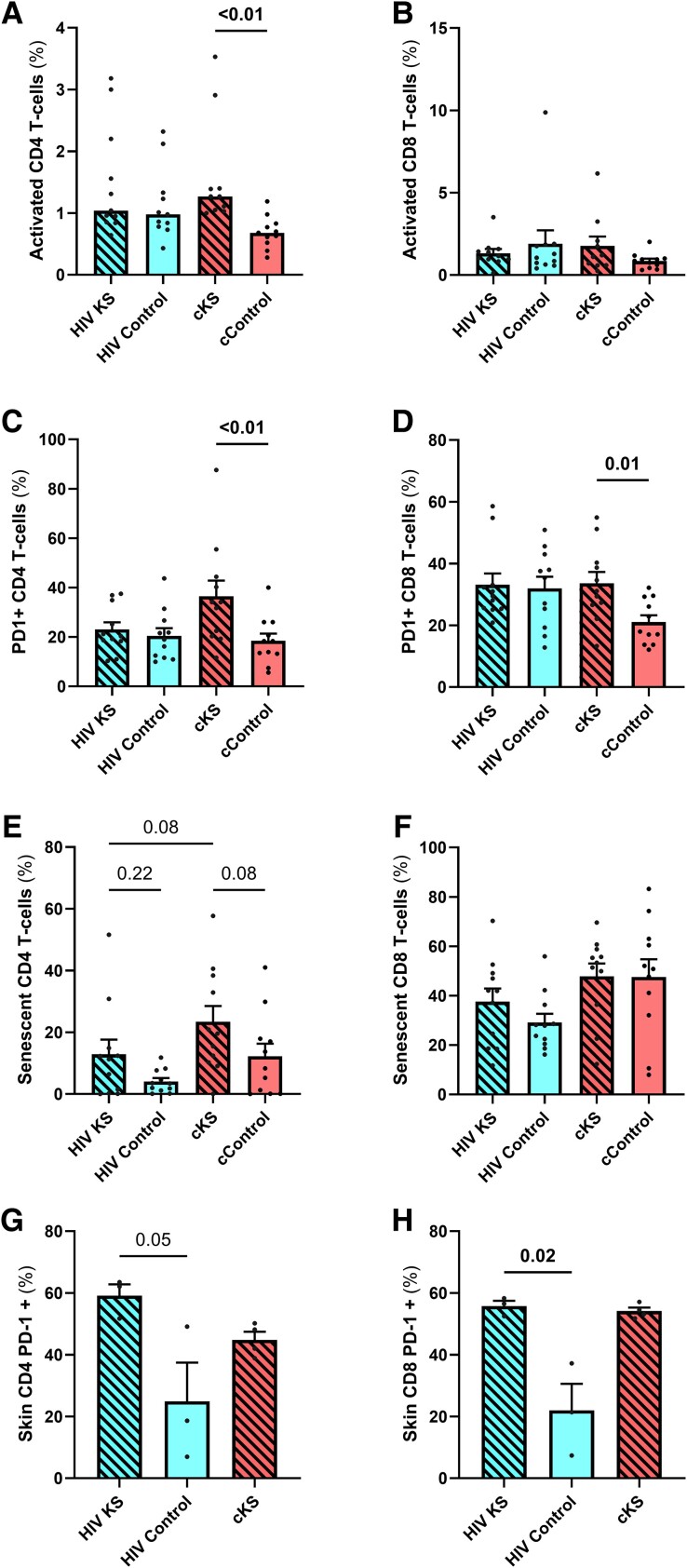
T-cells phenotypes in blood and skin biopsies. Blood CD4 (*A, C, E*) and CD8 (*B, D, F*) and skin CD4 (CD8–, *G*) and CD8 (*H*) T cells were phenotyped by flow cytometry. Activated T cells were selected as HLADR+CD38+, and CD28–CD57+ cells were considered senescent. Only significant (<.05) *P* values are indicated. cKS, classic KS; KS, Kaposi sarcoma.

We observed no difference in the frequency of activated CD4 T cells in PLWH with or without KS (*P* = .39), probably because of the higher levels of activated CD4 T cells in all HIV+ participants compared to controls (HIV+ vs HIV–: *P* = .03). cKS showed higher frequency of activated CD4 T cells (HLADR+CD38+) than cControls (*P* < .01, Figure 3*A*). No difference was observed between HIV KS and cKS. A similar trend with higher proportion of activated CD8 T cells in cKS compared to their cControls (*P* = .085, Figure 3*B*) was observed but not between HIV KS and HIV controls (*P* = .77) nor between HIV KS and cKS.

HIV KS and HIV controls had similar frequencies of CD4 T cells expressing PD-1 (*P* = .56). cKS had higher frequency of PD-1+ CD4 T cells compared to cControls (*P* < .01, Figure 3*C*). All groups had higher frequencies of PD-1 expressing CD8 T cells compared to cControls (*P* = .01, .02, and .03 respectively, Figure 3*D*). HIV KS and cKS had similar levels of PD-1 expressing CD4 and CD8 T cells.

HIV KS had slightly higher levels of senescent (CD28–CD57+) CD4 T cells compared to HIV controls, although not significant (*P* = .22). Also, cKS had a higher frequency of senescent CD4 T cells compared to cControls (*P* = .08, Figure 3*E*). Interestingly, frequency of senescent CD4 T cells tended to be lower in HIV KS compared to cKS (*P* = .08). Higher levels of senescent CD8 T cells were observed in cKS and cControl compared to HIV KS or HIV controls (HIV+ vs HIV–: *P* = .04, Figure 3*F*).

In cells isolated from skin lesions, the percentage of PD-1+ CD4 T cells was similar between HIV KS and cKS (*P* > .99, Figure 3*G*) and was higher than in HIV controls (*P* = .20 and .05, respectively). Moreover, frequency of PD-1 expressing CD8 T cells observed in HIV KS and cKS was similar (*P* = .61, Figure 3*H*), with higher levels in HIV KS than HIV controls (*P* = .02).

### Plasmacytoid Dendritic Cells Were Activated and Expressed PD-L1 in cKS

**Figure 4.**
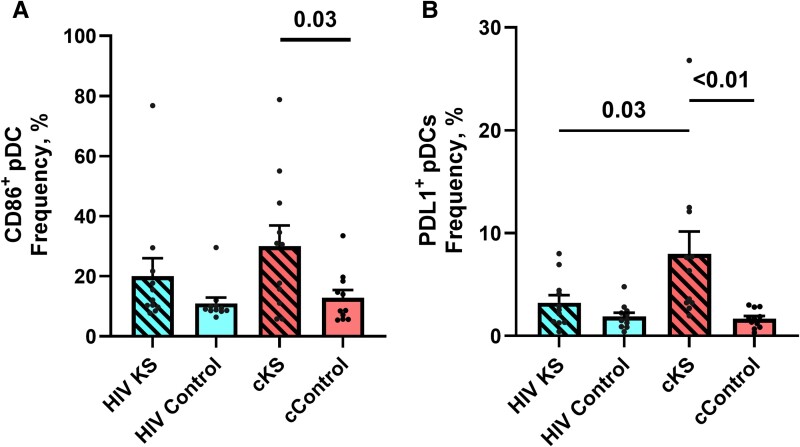
Dendritic cells phenotypes in blood. Blood conventional (HLADR+CD11c+) and plasmacytoid (CD11C–BDCA2+) dendritic cells were phenotyped for activation markers and immune-checkpoint ligands. (*A*) Frequency of activated (CD86+) pDCs. (*B*) Frequency of PDL1-expressing pDCs. Only significant (<.05) *P* values are indicated. cKS, classic KS; KS, Kaposi sarcoma.

In PBMCs, plasmacytoid dendritic cells (pDC), the DC subpopulation with strong antiviral properties, tended to be more activated (CD86+) in HIV KS compared to HIV controls (*P* = .17) and in cKS compared to their cControls (*P* = .03) (Figure 4*A*). Expression of PD-L1 on pDC was more frequent in cKS blood compared to all the other groups (cControls: *P* < .01; HIV KS *P* = .03; HIV controls: *P* < .01, Figure 4*B*).

Interestingly levels of CD86 and PD-L1 expression on pDC mirrored the levels of activation and PD-1 expression on CD4 T cells.

### Differences in Circulating Cytokine and Biomarkers Levels

**Figure 5.**
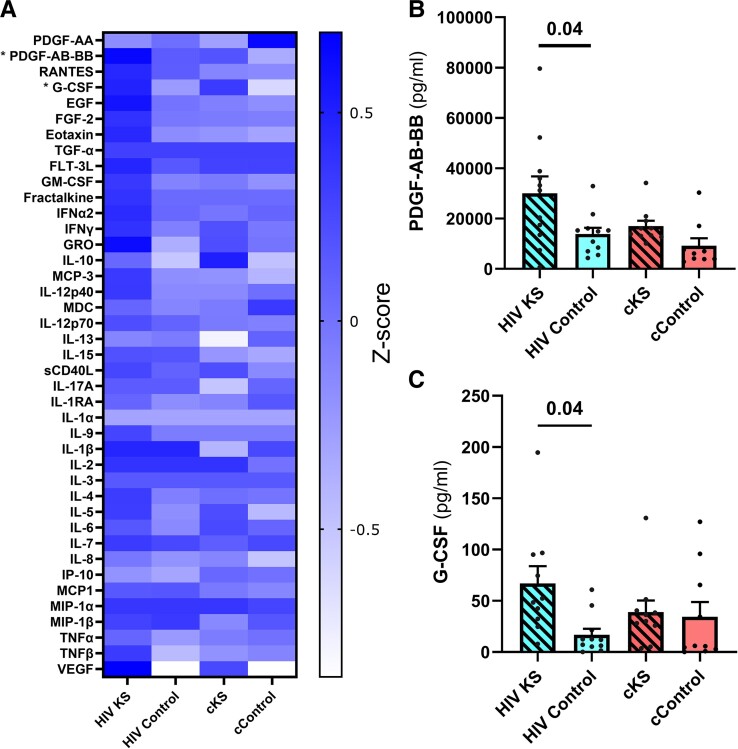
Plasma levels of cytokines, chemokines, and growth factors. Using multiplexes, we quantified 41 cytokines, chemokines, and growth factors in the plasma of the 44 participants. (*A*) Heatmap *Z*-scores of plasma levels of each analyte. Significant differences were observed for PDGF-AB-BB (*B*) and G-CSF (*C*) using the Kruskal-Wallis test. Only significant (<.05) *P* values are indicated. cKS, classic KS; KS, Kaposi sarcoma.

Various levels of cytokines and growth factors were observed between groups (Figure 5*A*, Supplementary Table 5). Kruskal-Wallis test indicated significant higher levels of 2 cytokines in HIV KS compared to HIV controls: (1) the platelet-derived growth factors (PDGF) AB-BB with (*P* = .04, Figure 5*B*) and (2) the granulocyte colony-stimulating factor (*P* = .04, Figure 5*C*).

## DISCUSSION

We found that PLWH with KS were younger than HIV-negative participants with cKS while exhibiting similar clinical presentations, and a lower CD4/CD8 ratio as a surrogate marker of chronic inflammation and risks of non-AIDS comorbidities, in line with previous findings [1, 2, 7, 14]. Interestingly, the CD4/CD8 ratio was not significantly different between cKS and HIV controls. All PLWH and most PwoH were seropositive for EBV and CMV. Whether CD8 T-cell expansion in HIV KS was a risk factor for KS, and was induced by HHV-8 replication, EBV or CMV remains to be determined [7]. Of note, we found a low frequency of anti-HHV8 T cells in KS patients. Despite a 20-year difference, we found similar composition of the circulating and lesion-infiltrating immune cells.

HHV-8 and HIV can interact through regulatory Tat and Nef proteins or by exosomes [15, 16]. A consistent trend of increased number of HIV blips and transcriptionally active reservoirs was found in PLWH with KS. HIV or its proteins might thus play a role in KS development by transactivation of virus production, in association with a dysfunctional CD8 cytotoxic activity and antibody production. Those findings highlight the importance of optimal and early ART initiation [17].

Although HHV-8 viral loads were similar in tissues among the 2 KS groups, circulating levels of HHV-8 DNA (both in plasma and isolated PBMCs) were significantly lower in HIV KS than in cKS. A potential explanation for this observation is the higher anti–HHV-8 antibody levels found in HIV KS compared to cKS, which could neutralize circulating HHV-8 viruses but have a lower impact in tissues [18, 19]. This hypothesis should be explored in further studies. These observations highlight potential interest of vaccination strategies against HHV-8. However, no significant difference could be found between groups for the frequency of HHV-8–specific T cells, although this response was low, as previously reported, and remains ill-defined. Yet immune escape mechanisms of HHV-8 might be involved, notably with K3 and K5 HHV-8 membrane proteins removing major class I histocompatibility complexes from the cell surface [20] and HHV-8-Ox2 protein blocking antigen-specific T-cell response [21]. An additional gradual dysfunction of the immune system with aging or HIV-induced immunosenescence could also account for the progressive loss of HHV-8–specific T cells. Complex interactions between HHV-8 and EBV are also reported, especially in primary effusion lymphoma, where EBV increases KS genome maintenance [22]. Because we found a similar differences in anti–HHV-8 and anti-EBV IgG levels, such interactions should be further studied.

To assess the influence of T-cell function on KS development, we analyzed the phenotype of circulating and tissular T-cells. We found higher levels of activation markers on blood T cells (mostly CD4 compared to CD8 T cells) from cKS compared to their age-matched controls, which might reflect the immune response to HHV-8 infection. Among myeloid cells, pDCs were also more activated in participants from the 2 KS groups compared to their respective controls. However, we did not observe a difference between the 2 KS groups. The frequency of immunosenescent CD4 and CD8 T cells was similar between groups. In contrast, PD-1 expression on CD4 T cells and PD-L1 expression on pDCs compared to controls was significantly increased only in cKS but not in HIV KS. As expected, high levels of PD-1 expression were observed in controls with HIV for both CD4 and CD8 T cells. Hence, although observed at different age, immune senescence appeared at similar levels on T cells from the 2 KS groups, suggesting early immunosenescence in HIV KS. However, PD-1 overexpression in circulating T cells only characterized cKS.

One of the strengths of our study is the unique access to skin biopsies of KS lesions. Although only a few T cells could be isolated from those tissues (limiting the number of analyses), we could find that these cells harbored a high frequency of PD-1 expression compared to the skin of HIV controls, possibly resulting from the tumor microenvironment. High PD-1 expression has been associated with poor infiltration of antigen-specific cells and low tumoral cell elimination [23]. Unfortunately, we could not perform skin biopsies in cControls.

The limitation of our study lies on the limited number of participants because of the low prevalence of these types of KS. Moreover, we were not able to assess the influence of nadir CD4 count on our study parameters. Finally, viral and immune characteristics associated with KS should be assessed in a group of older HIV-uninfected people being seropositive for HHV-8. A multicentric collaborative study including more participants would thus be needed to confirm and expand our findings.

## CONCLUSIONS

Altogether, PLWH with KS harbor similar levels of immune activation and immune senescence than 20-year-older cKS, highlighting accelerated/accentuated aging in ART-treated PLWH. Specific characteristics of KS in PLWH still need to be addressed in basic and clinical research.

Although different on circulating T cells, an upregulation of PD-1 expression CD4 and CD8 T cells was observed in lesions of both KS groups. Together with higher PDGF-AA-AB and GCSF plasma levels in HIV KS, these observations foster relevance for PD-1 inhibitors and antiangiogenic drugs currently being tested to optimize therapeutic interventions for KS [3]. With the advent of condom-free preexposure prophylaxis and aging of PLWH, HHV-8–related diseases might become more frequent and will represent a growing therapeutic challenge [24].

## Supplementary Material

ofae404_Supplementary_Data

## Supplementary Data

Supplementary materials are available at *Open Forum Infectious Diseases* online. Consisting of data provided by the authors to benefit the reader, the posted materials are not copyedited and are the sole responsibility of the authors, so questions or comments should be addressed to the corresponding author.

## Notes

***Acknowledgments*.** The authors are grateful to Josée Girouard and Angie Massicotte for the nursing and administrative support, respectively, and to the personnel of the RI MUHC platforms, notably Marie-Helene Lacombe, Helene Pagé-Veillette, and Ekaterina Iourtchenko from the immunophenotyping platform. The following reagent was obtained through the NIH HIV Reagent Program, Division of AIDS, NIAID, NIH: Human herpesvirus 8 (HHV-8) Infected Body Cavity Based Lymphoma 1 (BCBL-1) cells, ARP-3233, contributed by Dr. Michael McGrath and Dr. Don Ganem.

***Financial support*.** Our work was funded by the Fonds de la Recherche Québec-Santé (FRQ-S): Réseau SIDA/Maladies Infectieuses and Thérapie Cellulaire; the Canadian Institutes of Health Research (CIHR; Grants HOP 103230, PTJ 166049, and DC0190GP); the Vaccines and Immunotherapies Core of the CIHR Canadian HIV Trials Network (CTN; Grant CTN 247); the Canadian Foundation for AIDS Research (CANFAR; Grant 02-512); the CIHR-funded Canadian HIV Cure Enterprise (CanCURE) Team Grant HB2-164064. L. R. is a postdoctoral fellow supported by the “Fonds de perfectionnement” of the Geneva University Hospitals, Switzerland, the Swiss National Science Foundation, and the CIHR/CTN. S. I. is a postdoctoral fellow supported by the FRQ-S and CIHR/CTN. J.-P. R. is the holder of the Louis Lowenstein Chair in Hematology and Oncology, McGill University.

***Author contributions*.** L. R., A. J., T. M., J. L., A. P., and S. I. performed measurements, acquired, and analyzed data, with input from C. A. B., I. L., N. C., A. C., V. L., V. C., A. G. M., and J. P. R. L. R. and J. P. R. recruited participants. L. R. and S. I. wrote the initial manuscript. All authors validated the submitted version.

***Patient consent statement*.** The authors confirm that written consent was obtained from all participants. The study has been approved by the Research Ethics Board of the McGill University Health Centre (REB # 2019-5170).
