# Supplementary material for: Similar Viral and Immune Characteristics of Kaposi Sarcoma in ART-treated People Living With HIV and Older Patients With Classic Kaposi Sarcoma
Source: Open Forum Infect Dis. 2024 Jul 12;11(8):ofae404. doi: 10.1093/ofid/ofae404 (PMC11295207; doi:10.1093/ofid/ofae404)
Supplement: ofae404_Supplementary_Data [file ofae404_supplementary_data.docx]

Similar viral and immune characteristics of Kaposi sarcoma in ART-treated people living with HIV and older patients with classic Kaposi sarcoma.

**Supplementary methods:**

**HHV8 detection in plasma, PBMCs and tissues.**

Plasma viral RNA/DNA, DNA from PBMC or skin tissues were extracted using commercial kits (Qiagen, Toronto, ON, Canada) and kept at –80°C until used. Plasma RNA/DNA was used directly. Prior to ddPCR, genomic DNA from PBMC or skin tissues were subjected to DNA digestion to increase droplet formation: 1µg of DNA was incubated with 800 U/mL of HindIII enzyme (New England BioLabs Inc. Whitby, ON, Canada) in the supplied buffer at 37°C for 1 hour, followed by heat inactivation of the enzyme at 80°C for 20 minutes according to supplier’s instructions.

Digital droplet PCR was performed according to supplier’s instructions (BioRad Laboratories, Montreal, QC, Canada). A set of primers and probe targeting HHV-8 ORF9 already published and validated has been used (**Supplementary Table 1**) [1]. Copies of HHV-8 were normalized using copies of RRP30 divided by 2 (as 2 copies of the gene are present per cell). Viral loads obtained with our ddPCR assay from PBMC and skin tissues were compared to viral loads quantified with a published and validated real-time qPCR assay [2].

**HHV8 genotyping**

Briefly, after target enrichment according to the SeqCap EZ HyperCap Workflow (Roche, Roche Diagnostics Corporation, Indianapolis, Indiana, USA), pooled DNA library was sequenced with paired-end reads on the NextSeq 500 Illumina system to achieve a depth sequencing of 100X. HHV-8 genome assembly and consensus sequence were generated after trimming of the reads (Qscore > 30) by mapping and *de novo* assembly, as previously reported [3]. For half of them, sequencing failed due to a low HHV-8 viral load and ORF-K1 (or VR1) Sanger sequencing was performed [3]. All the sequences were submitted to the NCBI GenBank (<https://www.ncbi.nlm.nih.gov/genbank/>) to be freely accessible (**Supplementary Figure 6**).

**Serology assay**

BCBL1 cells were kept in culture in DMEM (Wisent Inc, Montreal, Qc, Canada) containing 20% FBS (Wisent), 100U/mL of penicillin and 100 µg/mL of streptomycin. BCBL-1 cells were stimulated for 48 hours with phorbol 12-myristate 13-acetate (Millipore MA, USA) at 20 ng/mL. Cells were washed and plated at 300.10^5^ cells per well in U-bottom 96 well plates in 100 µL of PBS containing 1mM EDTA (Thermofisher) and 2% FBS. 100uL of plasma or PBS (Wisent Inc, Montreal, Qc, Canada) as control was added, and mixed. Plates were incubated at 37°C for 1 hour. Cells were washed in PBS containing EDTA and FBS, then PBS. A volume of 100 µL of anti-human IgG (heavy and light chains) conjugated to Alexa-Fluor488 (Thermofisher cat #A-11013) diluted in PBS containing EDTA and FBS was added at 5 µg/mL 20 min at 37°C. Cells were then washed once in PBS, and incubated in 50 µL containing viability dye LiveDead-Blue (ThermoFisher) for 20 min at 4°C. Cells were then washed, fixed in PBS containing 2% paraformaldehyde (ThermoFisher). Cytometry acquisition was performed on a BD-Fortessa X20, analysis was performed using FlowJo version 10.9. Percentage of live BCBL1 positive for Alexa-Fluo488 was calculated.

**Supplementary Tables:**

**Supplementary Table 1. Primers and probe used for the HHV-8 ddPCR assay.** Based on [1], reference sequence GenBank AP017458.1.

|  | **Sequence (5′-3′)** | **Target** |
| --- | --- | --- |
| **Primers** | | |
| *HHV-8-Forward* | ATATACGGCGACACTGACTC | 13603 - 13623 |
| *HHV-8-Reverse* | GAGCAGAAGGCACTTGAAG | 13742 - 13761 |
| **Probe** | | |
| *HHV-8-Probe* | 56-FAM/CGGAGGAGC/ZEN/TAGCGTCAATCA/3IABkFQ | 13673 - 13693 |

**Supplementary Table 2**. Flow cytometry antibodies

| Skin phenotyping panel | | | |
| --- | --- | --- | --- |
| **Target** | **Labelling** | **Supplier** | **Cat number** |
| LiveDead | blue (UV) | Thermofisher | L-34962 |
| CD45 | A700 | Biolegend | 301840 |
| CD3 | BV711 | Biolegend | 300464 |
| CD8 | BUV737 | BD Biosciences | 612754 |
| CD45RA | APCCy7 | Biolegend | 304128 |
| CD19 | FITC | BD Biosciences | 555412 |
| HLADR | BV785 | Biolegend | 307642 |
| CD38 | BV605 | BD Biosciences | 562665 |
| PD-1 | BV421 | Biolegend | 329919 |
| LAG3 | PE | Biolegend | 369306 |
| TIGIT | PerCP Efluor 710 | Ebiosciences | 46-9500-42 |
| CD57 | BV510 | Biolegend | 393314 |

| PBMC T-cells | | | |
| --- | --- | --- | --- |
| **Target** | **Labelling** | **Supplier** | **Cat number** |
| LiveDead | blue (UV) | ThermoFischer | L23105 |
| CD3 | BV711 | Biolegend | 300464 |
| CD4 | BUV395 | BD BioSciences | 562724 |
| CD8 | BUV737 | BD Biosciences | 612754 |
| CD45RA | APCCy7 | Biolegend | 304128 |
| CD19 | FITC | BD Biosciences | 555412 |
| CD25 | A700 | Biolegend | 356118 |
| HLADR | BV785 | Biolegend | 307642 |
| CD38 | BV605 | BD Biosciences | 562665 |
| PD-1 | BV421 | Biolegend | 329919 |
| CTLA4 (CD152) | APC | Biolegend | 349908 |
| LAG3 | PE | Biolegend | 369306 |
| CD57 | BV510 | Biolegend | 393314 |
| CD28 | PE-Cy7 | Biolegend | 302926 |

| PBMC Monocytes/DC | | | | |
| --- | --- | --- | --- | --- |
| **Target** | **Labelling** | **Supplier** | **Cat number** |  |
| LIveDead | Blue (UV) | ThermoFischer | L23105 |  |
| CD3 | FITC | BD | 555332 |  |
| CD19 | FITC | BD | 555412 |  |
| CD56 | AF488 | BD | 561905 |  |
| CD16 | A700 | Biolegend | 302026 |  |
| HLA-DR | APC-Cy7 | BD | 335796 |  |
| CD14 | BV786 | Biolegend | 301839 |  |
| CD11c | BV711 | BD | 563130 |  |
| BDCA2 | APC | Biolegend | 314408 |  |
| PD-L1 (CD274) | PECy7 | Biolegend | 329717 |  |
| CD155 | PE | Biolegend | 337610 |  |
| CD80 | BV421 | Biolegend | 305222 |  |
| CD86 | PE/Dazzle 594 | Biolegend | 305433 |  |
| CD40 | PerCP-eFluor710 | eBioscience | 46-0409-42 |  |
| CCR7 (CD197) | BV510 | BD | 563449 |  |

**Supplementary Table 3.** HHV-8 genotypes obtained by sequencing and typing for each participant with KS, based on K1 sequence homology.

| **ID** | **Group** | **Sample** | **Sequencing** | **Genotype based of K1** |
| --- | --- | --- | --- | --- |
| HP2 | KS HIV- (inuit) | Skin1 | NGS | new variant D |
| HP9 | KS HIV- | PBMC1 | both failed | - |
| HP16 | KS HIV+ | Skin1 | NGS | C |
| HP17 | KS HIV- | plasma | ND | ND |
| HP21 | KS HIV- | Skin1 | NGS | C3 |
| HP27 | KS HIV- | PBMC1 | sanger K1 | A5 |
| HP28 | KS HIV+ | PBMC1 | both failed | - |
| HP43 | KS HIV- (inuit) | PBMC1 | sanger K1 | new variant D |
| HP49 | KS HIV+ | Skin1 | NGS | C |
| HP50 | KS HIV+ | plasma | ND | ND |
| HP56 | KS HIV+ | PBMC1 | sanger VR1 | A5 |
| HP60 | KS HIV- | plasma | ND | ND |
| HP61 | KS HIV+ | Skin2 | NGS | C3 |
| HP79 | KS HIV+ | PBMC2 | sanger VR1 | A |
| HP83 | KS HIV+ | PBMC1 | sanger VR1 | A |
| HP102 | KS HIV+ | Skin1 | NGS | A5 (B by NGS) |
| HP124 | KS HIV- | Skin1 | NGS | C3 |
| HP126 | KS HIV- | Skin1 | NGS | B (F1 by NGS) |

Genbank accession number: 1-HP16-Skin1: OQ102640, 2-HP16-Skin3: OQ102644, 3-HP126-Skin1: OQ102652, 4-HP124-Skin1: OQ102641, 5-HP2-Skin1: OQ102650, 6-HP102-Skin1: OQ102648, 7-HP21-Skin1: OQ102645, 8-HP58-PBMC1: OQ102649, 9-HP16-Skin1: OQ102643, 10-HP49-Skin1: OQ102647, 11-HP27-Skin1: OQ102646, 12-HP61-Skin1: OQ102642, 13-HP9-PBMC1: OQ102651

**Supplementary Table 4. HHV-8 peptides for T-cell assay, based on** [6,7]

| HHV-8 peptides for T-cell assay | |
| --- | --- |
| **Pepmix HHV-8 K12** | MDRGLTVFVAVHVPDVLLNGWRWRLGAIPPLVCLLAISVVPPSGQRGPVAFRTRVATGAH |
| **Peptide pool LANA1** | LANA-1_238–246_ WATESPIYV |
|  | LANA-1_281–289_ AMLVLLAEI |
|  | LANA-1_78–86_ FVSSPTLPV |
|  | LANA-1_1116–1124_ QMARLAWEA |
|  | LANA-1_71–79_ FTSGLPAFV |
|  | LANA-1_1120–1128_ LAWEASHPL |
|  | LANA-1_285–293_ LLAEIAEEA |
|  | LANA-1_1135–1143_ SIVKFKKPL |
|  | LANA-1_61–70_ TLPIPGSPTV |
|  | LANA-1_140-148_ PESSQRPPL |
|  | LANA-1_417-425_ EDKKEDEED |
|  | LANA-1_688-697_ EQEQELEEQE |
|  | LANA-1_920-628_ PPGDNTPDD |
| **Peptide pool K8.1** | K8.1_73-81_ RLAAGSPSS |
|  | K8.1_135-143_ ALISAFSGS |
|  | K8.1_209-217_ LVLILYLCV |
| **Peptide pool gpB** | gpB_492–500_ LMWYELSKI |
|  | gpB_736-745_ MLMIIIVIAI |

**Supplementary Table 5: multiplex results in plasma**

| Pg/mL | HIV KS | | | HIV control | | | cKS | | | cControl | | |  | LLOD |
| --- | --- | --- | --- | --- | --- | --- | --- | --- | --- | --- | --- | --- | --- | --- |
|  | Median | Max | Min | Median | Max | Min | Median | Max | Min | Median | Max | Min |  |  |
| PDGF-AA | 2675 | 8643 | 385 | 3676 | 37943.75 | 545.84 | 2185 | 39068.99 | 412 | 11067 | 26481.04 | 1286 |  | 0.02 |
| PDGF-AB-BB | 31197 | 79619 | 660 | 13911 | 34171 | 0 | 14945.17 | 32899 | 4333 | 6524 | 141200 | 2821.29 |  | 0.15 |
| RANTES | 58848 | 123300 | 2947 | 41274 | 215548 | 1006 | 31525 | 74197 | 7544 | 30369 | 393000 | 14773 |  | 0.32 |
| G-CSF | 48.44 | 194.59 | 0 | 11.97 | 60.81 | 0 | 36.42 | 21836 | 3.57 | 5.84 | 127.01 | 0 |  | 0.086 |
| EGF | 55.48 | 144.25 | 9.54 | 23.83 | 48.47 | 0 | 21.36 | 72.02 | 5.93 | 19.13 | 74.94 | 8.31 |  | 3.2 |
| FGF-2 | 85.83 | 188.46 | 14.42 | 65.98 | 131.01 | 12.5 | 64.9 | 146.59 | 41.81 | 65.11 | 453.74 | 28.97 |  | 1.09 |
| Eotaxin | 156.74 | 288.16 | 95.04 | 122.77 | 190.23 | 46.98 | 120.32 | 220.9 | 56.32 | 116.41 | 245.51 | 90.21 |  | 1.6 |
| TGF-a | 0 | 28.4 | 0 | 0 | 1.84 | 0 | 0 | 0.42 | 0 | 0 | 33.76 | 0 |  | 0.01 |
| FLT-3L | 39.49 | 98.63 | 17.33 | 23.52 | 45.48 | 0 | 29.23 | 643.63 | 0 | 29.47 | 232.97 | 0 |  | 0.24 |
| GM-CSF | 6.31 | 35.39 | 0 | 3.44 | 17.68 | 0 | 3.7 | 26.29 | 0.51 | 3.31 | 173.54 | 0 |  | 0.11 |
| Fractalkine | 108.41 | 794.6 | 0.96 | 74.16 | 184.56 | 26.14 | 74.16 | 187.33 | 38.1 | 73.59 | 873.33 | 3.06 |  | 1.46 |
| IFNa2 | 26.39 | 66.26 | 1.2 | 15.98 | 73.89 | 0 | 14.29 | 47.02 | 4.82 | 17.22 | 401.31 | 0.12 |  | 0.54 |
| IFNg | 10.02 | 51.74 | 0 | 5.37 | 25.2 | 0.52 | 7.87 | 33.38 | 0.51 | 5.69 | 199.36 | 0 |  | 0.32 |
| GRO | 2138 | 3752 | 502.26 | 929.68 | 3767 | 101.71 | 1521 | 3285 | 335.46 | 1345 | 3807 | 273.69 |  | 0.51 |
| IL-10 | 7.77 | 22.22 | 3.05 | 4.66 | 22.15 | 1.02 | 11.66 | 83.39 | 3.06 | 4.79 | 21.64 | 2.91 |  | 0.05 |
| MCP-3 | 28.16 | 42.13 | 14.99 | 19.28 | 182.27 | 6.17 | 18.95 | 38.61 | 10.97 | 16.59 | 168.38 | 3.31 |  | 1.6 |
| IL-12p40 | 9.27 | 20.61 | 2.12 | 5.01 | 71.22 | 0 | 5.01 | 32.67 | 1.23 | 6.77 | 218.61 | 0 |  | 0.3 |
| MDC | 559.61 | 806.28 | 340.93 | 506.65 | 766.78 | 255.4 | 523.07 | 2985 | 72.71 | 639.95 | 1107 | 246.38 |  | 0.47 |
| IL-12p70 | 2.59 | 89.49 | 0.01 | 2.14 | 8.23 | 0 | 1.73 | 6.32 | 0.21 | 1.67 | 123.27 | 0 |  | 0.32 |
| IL-13 | 0.65 | 20.65 | 0 | 0.78 | 73.24 | 0 | 0.2 | 6.23 | 0 | 0.95 | 122.62 | 0 |  | 0.06 |
| IL-15 | 1.95 | 6.06 | 0.62 | 1.88 | 19.1 | 0.01 | 0.97 | 56.36 | 0 | 0.82 | 66.45 | 0 |  | 0.02 |
| sCD40L | 334.15 | 1756 | 89.76 | 292.11 | 885.21 | 23.04 | 321.63 | 569.79 | 91.16 | 246.6 | 616.21 | 45.63 |  | 0.38 |
| IL-17A | 0.98 | 17.3 | 0 | 0.98 | 5.94 | 0 | 0.38 | 3.02 | 0.04 | 0.33 | 44.95 | 0 |  | 0.05 |
| IL-1RA | 27.49 | 63.93 | 6.54 | 23.09 | 57.12 | 6.71 | 23.99 | 97.82 | 14.86 | 29.31 | 224.46 | 7.32 |  | 0.21 |
| IL-1a | 0.31 | 53.27 | 0 | 0.25 | 189.44 | 0 | 0.68 | 16.43 | 0 | 0.51 | 363.85 | 0 |  | 0.07 |
| IL-9 | 1.82 | 7.57 | 0.06 | 0.01 | 78.72 | 0 | 0.59 | 4.18 | 0 | 0.51 | 133.43 | 0 |  | 0.32 |
| IL-1b | 0.9 | 11.82 | 0 | 0.19 | 4.79 | 0 | 0.28 | 2.02 | 0.02 | 0.32 | 48.65 | 0 |  | 0.03 |
| IL-2 | 0.01 | 5.82 | 0 | 0 | 2.5 | 0 | 0 | 0.87 | 0 | 0.02 | 43.36 | 0 |  | 0.32 |
| IL-3 | 0 | 0.05 | 0 | 0 | 0 | 0 | 0 | 0 | 0 | 0 | 0 | 0 |  | 0.02 |
| IL-4 | 39.88 | 70.44 | 3.36 | 18.08 | 782.16 | 0 | 21.98 | 87 | 6.02 | 21.44 | 1729 | 1.15 |  | 0.32 |
| IL-5 | 2.25 | 4.75 | 0.89 | 1.38 | 19.79 | 0.16 | 2.04 | 4.29 | 0.65 | 1.1 | 25.39 | 0.18 |  | 0.03 |
| IL-6 | 0.46 | 10.54 | 0 | 0.27 | 110.67 | 0 | 0.46 | 10.59 | 0 | 1.1 | 102.64 | 0 |  | 0.01 |
| IL-7 | 3.96 | 53.68 | 0.03 | 3.47 | 13.15 | 0 | 2.77 | 19.89 | 0 | 3.48 | 70.18 | 0 |  | 0.08 |
| IL-8 | 4.91 | 13.76 | 2.34 | 4.34 | 49.9 | 1.97 | 4.63 | 12.17 | 1.91 | 3.46 | 66.82 | 2.24 |  | 0.32 |
| IP-10 | 432.35 | 1095 | 179.56 | 402.66 | 1436 | 99.23 | 511.45 | 5074 | 146.38 | 496.22 | 842.29 | 316.82 |  | 0.28 |
| MCP1 | 250.07 | 713.79 | 27.95 | 252.25 | 336.02 | 108.03 | 219.27 | 1332 | 150.82 | 209.19 | 298.29 | 31.26 |  | 0.11 |
| MIP-1a | 0 | 4.64 | 0 | 0.01 | 12.38 | 0 | 0 | 11.73 | 0 | 0.02 | 25.21 | 0 |  | 0.03 |
| MIP-1b | 16.85 | 27.94 | 8.84 | 17.26 | 25.81 | 7.01 | 14.12 | 35.94 | 5.67 | 16.04 | 29.6 | 10.33 |  | 0.01 |
| TNFa | 13.8 | 22.61 | 9.26 | 11.85 | 17.12 | 9.88 | 12.9 | 60.78 | 5.14 | 13.51 | 52.11 | 5.6 |  | 0.04 |
| TNFb | 4.5 | 41.44 | 0.13 | 0.85 | 281.31 | 0 | 1.56 | 32.03 | 0.04 | 1.96 | 433.4 | 0 |  | 0.15 |
| VEGF | 25.55 | 368.44 | 0 | 0 | 70.3 | 0 | 10.44 | 100.18 | 0 | 0 | 501.22 | 0 |  | 2.11 |

cKS: classic KS; LLOD: lower limit of detection.

**Supplementary Figures:**

**Supplementary Figure 1**. T-cell phenotyping analysis gating strategy in PBMCs.


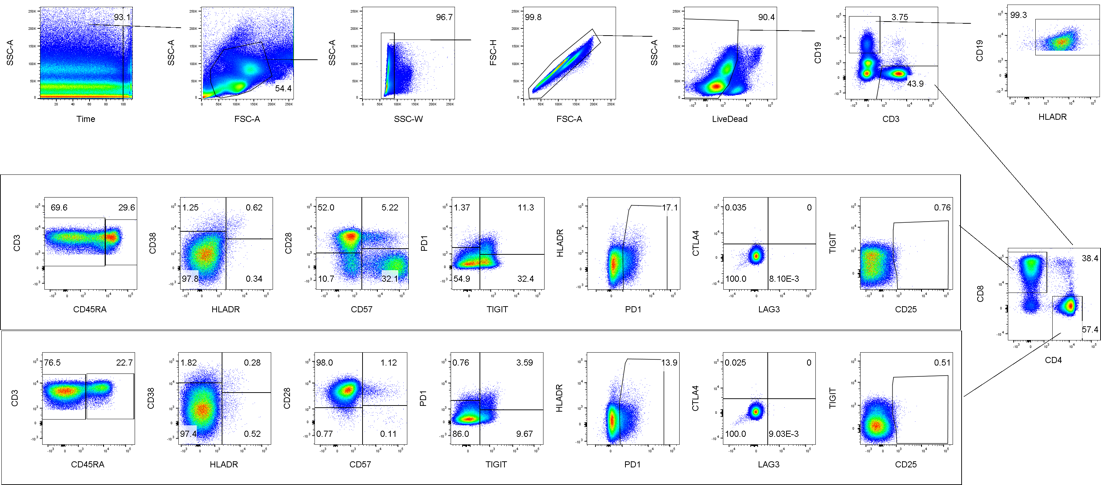


**Supplementary Figure 2**. Monocyte/DC phenotyping analysis gating strategy in PBMCs.


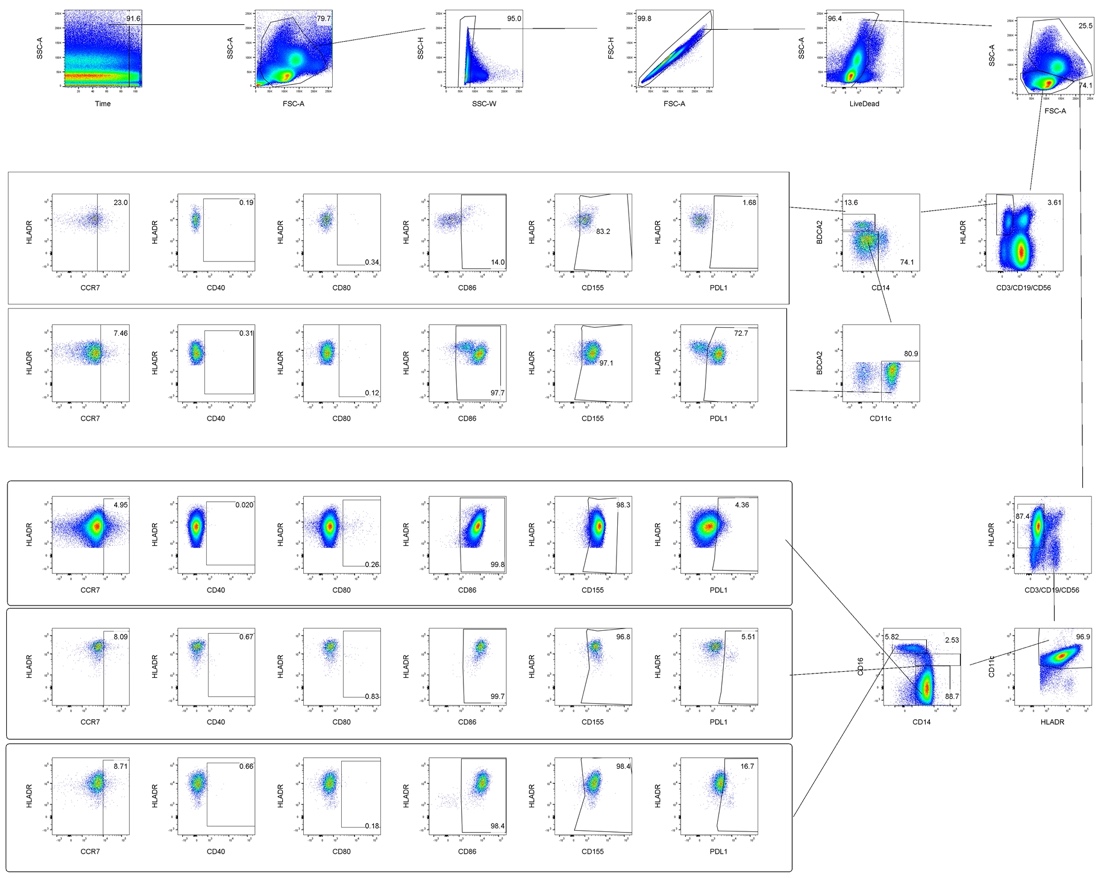


**Supplementary Figure 3**. Phenotyping of skin T cells


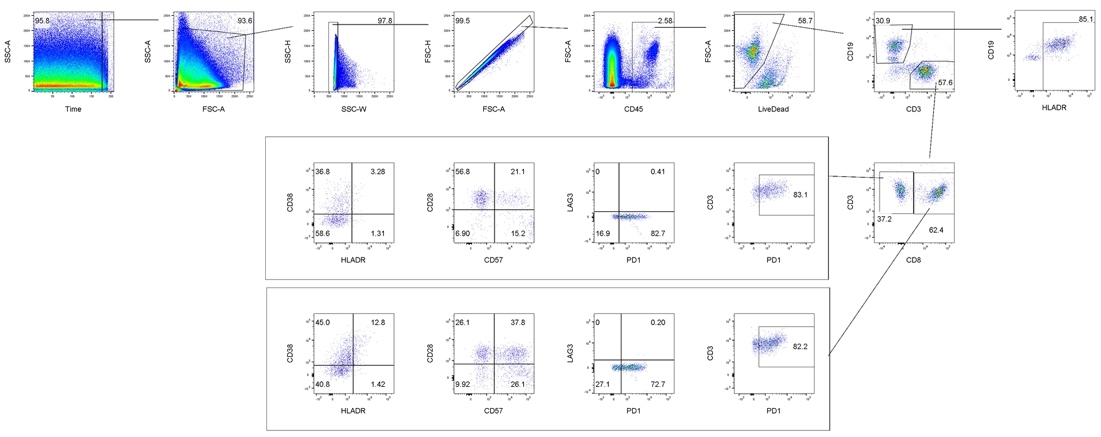

**Supplementary Figure 4**. KS skin lesions of HIV+ and HIV- participants. All participants with KS exhibited limited skin lesions predominantly on lower limbs.

**Supplementary Figure 5.** Comparison of HHV-8 viral loads obtained with our in-house ddPCR assay (y-axis) and a validated Taqman qPCR assay (x-axis).

To assess sensitivity and reliability of our ddPCR assay, we compared HHV-8 DNA quantification obtained with our in-house ddPCR assay and with a validated qPCR assay [2]. Viral loads in PBMC (square symbol) and skin biopsies (circle symbol) obtained with both assays strongly correlated (r=0.91, *p*<0.0001). As expected for ddPCR assays, our assay was more sensitive than qPCR to detect samples with low viral loads (<10^3^ copies/10^6^ cells, data not shown).

**Supplementary Figure 6. HIV reservoirs and blips.** Number of HIV blips (defined as HIV viral load > 20 copies/ml) per year (A) and per HIV viral load test (B) was quantified for participants living with HIV with or without KS. HIV reservoir size was estimated using total HIV DNA in CD4 T-cells (C), integrated HIV DNA in CD4 T-cells (D), HIV RNA in CD4 T-cells (E), ratio of HIV RNA compared to total DNA (F) and HIV RNA compared to integrated DNA (G) and compared for participants living with HIV with or without KS. Only significant (<0.05) *p*-values are indicated. KS: Kaposi sarcoma; HIV+: people living with human immunodeficiency virus; HIV-: uninfected controls; CD4^+^: CD4 T-cells.


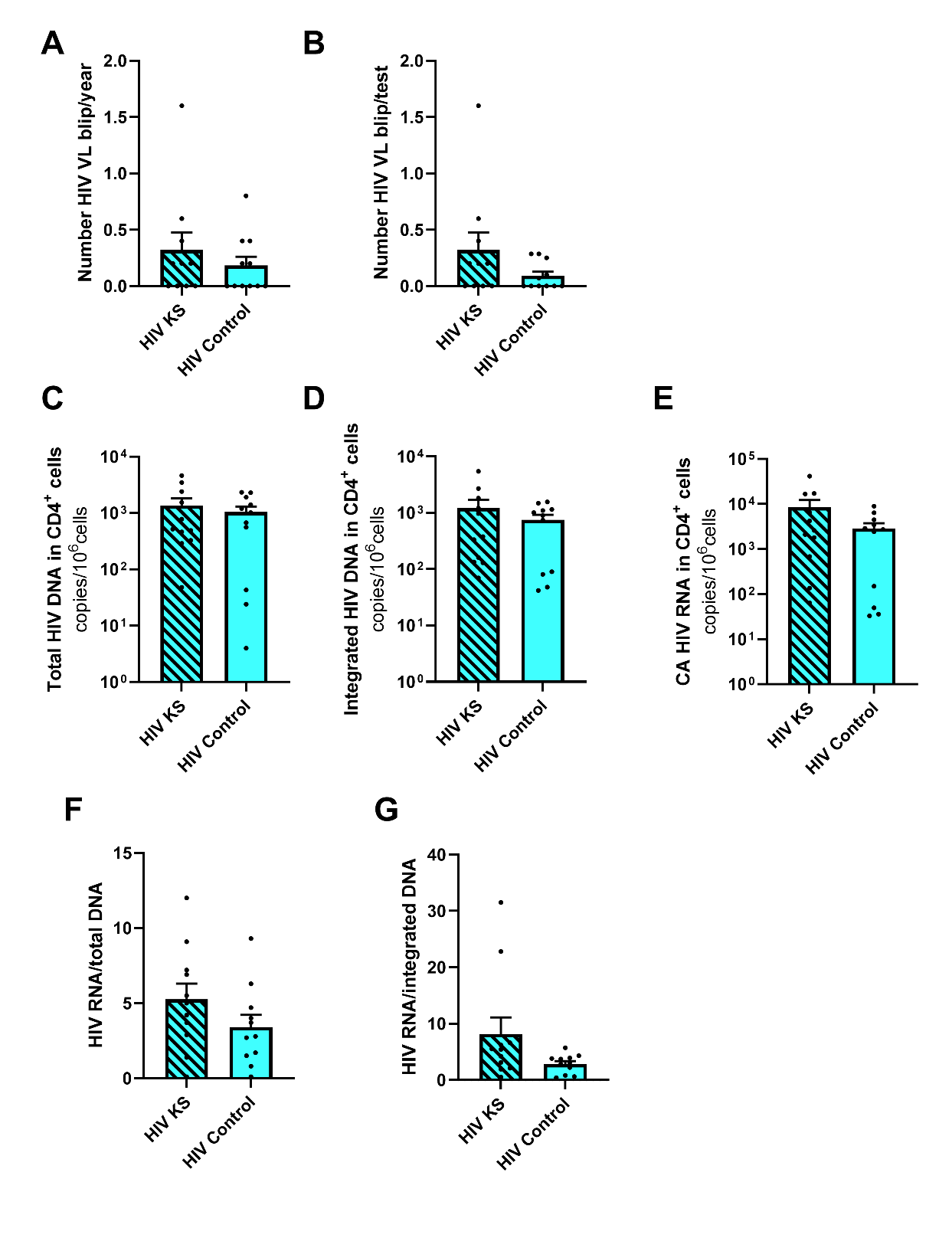


**Supplementary Figure 7**. IgG levels against EBV (A) and CMV (B) among all groups.


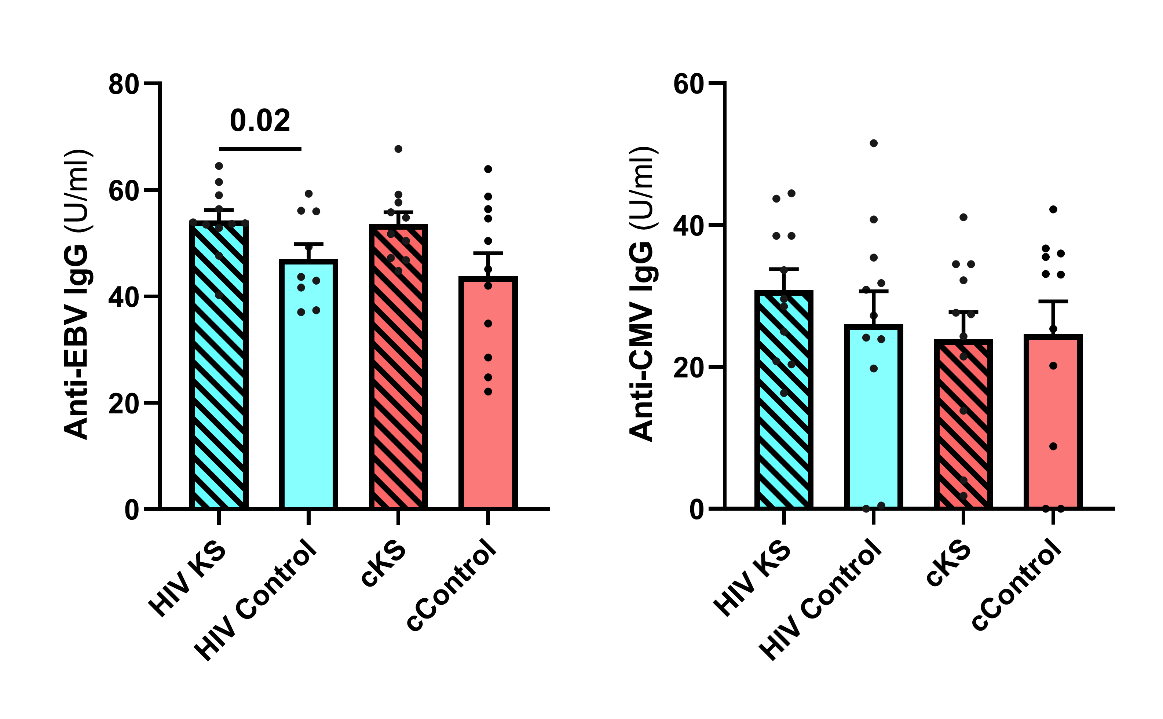


**B**

**A**

**BIBLIOGRAPHY**

1. Pyöriä, L.; Jokinen, M.; Toppinen, M.; Salminen, H.; Vuorinen, T.; Hukkanen, V.; Schmotz, C.; Elbasani, E.; Ojala, P.M.; Hedman, K.; et al. HERQ-9 Is a New Multiplex PCR for Differentiation and Quantification of All Nine Human Herpesviruses. *mSphere* **2020**, *5*, e00265-20, doi:10.1128/mSphere.00265-20.

2. Corgiat, M.; Calvez, V.; Marcelin, A.-G.; Jary, A. Methods Comparison for Molecular Diagnosis of Human Herpesvirus 8 Infections. *J Clin Virol* **2020**, *126*, 104308, doi:10.1016/j.jcv.2020.104308.

3. Jary, A.; Leducq, V.; Desire, N.; Petit, H.; Palich, R.; Joly, V.; Canestri, A.; Gothland, A.; Lambert-Niclot, S.; Surgers, L.; et al. New Kaposi’s Sarcoma-Associated Herpesvirus Variant in Men Who Have Sex with Men Associated with Severe Pathologies. *J. Infect. Dis.* **2020**, doi:10.1093/infdis/jiaa180.

4. Katoh, K.; Standley, D.M. MAFFT Multiple Sequence Alignment Software Version 7: Improvements in Performance and Usability. *Mol Biol Evol* **2013**, *30*, 772–780, doi:10.1093/molbev/mst010.

5. IQ-TREE: A Fast and Effective Stochastic Algorithm for Estimating Maximum-Likelihood Phylogenies - PubMed Available online: https://pubmed-ncbi-nlm-nih-gov.proxy3.library.mcgill.ca/25371430/ (accessed on 10 May 2023).

6. Guihot, A.; Dupin, N.; Marcelin, A.-G.; Gorin, I.; Bedin, A.-S.; Bossi, P.; Galicier, L.; Oksenhendler, E.; Autran, B.; Carcelain, G. Low T Cell Responses to Human Herpesvirus 8 in Patients with AIDS-Related and Classic Kaposi Sarcoma. *J Infect Dis* **2006**, *194*, 1078–1088, doi:10.1086/507648.

7. Nalwoga, A.; Roshan, R.; Moore, K.; Marshall, V.; Miley, W.; Labo, N.; Nakibuule, M.; Cose, S.; Rochford, R.; Newton, R.; et al. Kaposi’s Sarcoma-Associated Herpesvirus T Cell Responses in HIV Seronegative Individuals from Rural Uganda. *Nat Commun* **2021**, *12*, 7323, doi:10.1038/s41467-021-27623-8.
